# Enhancing Handover Quality and Continuity of Care: Implementation and Evaluation of a Digital Handover System in Grangewood Hospital, Northern Ireland

**DOI:** 10.1192/bjo.2025.10386

**Published:** 2025-06-20

**Authors:** Lewis Kitchen, Adam Flynn

**Affiliations:** Grangewood Hospital, WHSCT, Londonderry, United Kingdom

## Abstract

**Aims:** To improve the quality of the handover process among resident doctors at Grangewood Hospital, Northern Ireland, through a digital handover system, targeting universal adoption (100%) of electronic documentation.

**Methods:** A digital handover system was implemented and evaluated over two months. A standardised pro forma was designed to allow for structured documentation for new and existing inpatients, covering patient demographics, legal status, clinical history, provisional diagnosis, and a management plan, including any outstanding tasks. The document was securely uploaded daily to a designated Digital SharePoint, ensuring compliance with local General Data Protection Regulation (GDPR) mandates. The digital system functioned as a dynamic and editable document and was designed to supplement verbal handover.

Data collection focused on evaluating adherence to handover completion and the presence of key clinical details: patient demographics, provisional diagnoses, brief histories, and management plans, including outstanding clinical tasks. Given the absence of a formalised handover framework prior to implementation, baseline assessments concentrated on measuring compliance and data completeness.

A driver diagram identified key enablers for successful implementation, and a Plan-Do-Study-Act (PDSA) cycle supported iterative refinements. Two structured educational interventions at Weeks 1 and 4 reinforced engagement. Additional sessions after Week 2 addressed emerging challenges.

**Results:** Of 50 potential handover episodes, 42 were successfully completed. Compliance rates improved from 40% in Week 1 to 80% in the final week, with an overall mean compliance rate of 84% over the 10-week period. The completeness of handover documentation averaged 76.72%, with the following component-specific inclusion rates:

Patient demographics: 68.25%.

Provisional diagnosis: 74.76%.

Brief patient history: 82.29%.

Outstanding tasks: 80.98%.

An improvement in documentation quality was observed following the second-week educational intervention, highlighting the importance of structured training.

**Conclusion:** Continuity of care is central to medical practice, as outlined in Good Medical Practice (2023). The digital handover system enhanced accuracy, completeness, and consistency, benefiting patient safety and workflow efficiency. While compliance rates indicate engagement, sustained adherence depends on continued education and refinement. Future efforts should focus on optimising usability and embedding digital handover into routine clinical practice to ensure long-term adoption.

